# Evaluating the Diagnostic Utility of dd-cfDNA in Renal Allograft Surveillance: A Single-Center Perspective

**DOI:** 10.3390/genes16070724

**Published:** 2025-06-21

**Authors:** Aja Aravamudhan, Kira Krug, Michelle Stoffel, Penn Muluhngwi

**Affiliations:** Laboratory Medicine and Pathology, University of Minnesota, Minneapolis, MN 55455, USAkrugx046@umn.edu (K.K.); stoff129@umn.edu (M.S.)

**Keywords:** donor-derived cell-free DNA (dd-cfDNA), donor-specific antibody (DSA), transplant rejection, kidney and pancreas transplantation

## Abstract

**Background:** Donor-derived cell-free DNA (dd-cfDNA) testing offers a non-invasive approach for monitoring allograft health in transplant recipients. However, its diagnostic performance and clinical utility remain insufficiently characterized across diverse populations. **Objectives:** This study assesses concordance between dd-cfDNA, donor-specific antibody (DSA) testing, and biopsy, while also comparing two commercial assays (AlloSure and Prospera) in kidney and pancreas transplant recipients. **Methods:** We retrospectively analyzed 271 transplant patient records from 2019 to 2024 at our institution, focusing on dd-cfDNA testing. Statistical analyses evaluated assay performance in relation to DSA and biopsy results. The impact of multi-organ transplantation (MOT) on dd-cfDNA levels was also assessed. **Results:** In our predominantly Caucasian cohort (61.5%) with a mean age of 53 years, increased levels of dd-cfDNA were significantly associated with DSA positivity, particularly within the Prospera group (*p* = 0.002), and were particularly higher in patients with HLA class II DSA. Both assays showed a limited correlation with biopsy-confirmed rejection. AlloSure had high specificity (80%) but low sensitivity (19%), whereas Prospera showed higher sensitivity (75%) with moderate specificity (60%). Dd-cfDNA levels were elevated in MOT recipients in a vendor-dependent manner. **Conclusions:** Our findings highlight the differing clinical strengths of dd-cfDNA assays: AlloSure demonstrates greater preference for ruling out rejection, whereas Prospera appears better suited for early detection. Dd-cfDNA interpretation in MOT recipients warrants cautious consideration. Overall, tailoring assay selection and optimizing diagnostic thresholds to clinical context may enhance transplant surveillance and patient management strategies.

## 1. Introduction

Donor-derived cell-free DNA (dd-cfDNA) testing offers a non-invasive alternative to traditional biopsy for monitoring allograft health in solid organ transplant recipients [1]. Various clinical tools are currently used to assess graft function, including serum creatinine measurements in renal transplantation [2], therapeutic drug monitoring of immunosuppressants [3], detection of donor-specific antibodies (DSAs) [4], imaging modalities such as ultrasound [5], and organ biopsy [6], which, despite being the gold standard, remains the most invasive diagnostic option. In contrast to biopsy, dd-cfDNA assays are a lower-risk test with promise for the early detection of subclinical graft injury and routine post-transplant surveillance [1,7,8]. However, elevated dd-cfDNA levels may also result from non-rejection causes such as infection or non-specific cellular injury, limiting specificity [9,10].

Dd-cfDNA consists of short DNA fragments (~166 base pairs) released from donor organ cells into the recipient’s bloodstream and other body fluids [11]. Due to their low abundance, detecting these fragments requires highly sensitive methodologies, typically advanced polymerase chain reaction (PCR) platforms or next-generation sequencing (NGS). When plasma is the sample matrix, it is collected either in standard potassium-EDTA tubes or preferably in specialized Streck cfDNA-preserving tubes. These tubes are designed to inhibit genomic DNA contamination from blood cell lysis, reduce interference from heparin, and maintain cfDNA stability for up to seven days at room temperature (18–22 °C) [12,13].

At our institution, dd-cfDNA testing is implemented as a send-out service via two commercial vendors, and, when clinically indicated, used alongside DSA testing and biopsy as part of routine transplant surveillance. Both the AlloSure (CareDx, Brisbane, CA, USA) and Prospera (Natera, San Carlos, CA, USA) assays—each based on next-generation sequencing (NGS)—are employed, with vendor selection determined by clinician preference. AlloSure uses 266 single-nucleotide polymorphisms (SNPs) to accurately quantify dd-cfDNA in transplant recipients without separate genotyping of donor or recipient [14,15] while Prospera uses more than 13,000 single-nucleotide polymorphisms (SNPs) and advanced bioinformatics to differentiate recipient and donor cfDNA [16,17].

While the clinical value of dd-cfDNA assays has been explored in multiple transplant cohorts [2,14,16,18], their diagnostic performance is still not fully comprehended within our patient population—especially among recipients of renal and pancreas transplants, including multi-organ transplant (MOT) recipients. This retrospective study aims to assess concordance between dd-cfDNA results and established diagnostic methods, including biopsy and DSA, over a five-year period. Furthermore, we aim to compare the clinical utility of the AlloSure and Prospera assays to assess their respective roles in our setting. These findings may inform future decisions about the feasibility of developing in-house dd-cfDNA testing and identifying patient subgroups best suited for each of these testing platforms.

## 2. Materials and Methods

### 2.1. Data Collection and Division into Subgroups

A total of 271 patient records were retrieved from the electronic health records (EHR) system (EPIC) at the University of Minnesota Medical Center. These records corresponded to patients who underwent dd-cfDNA testing between January 2019 and February 2024. Each record was individually reviewed to verify patient consent for data usage for research purposes; only those with documented informed consent were included. Records with missing or unreliable test results were excluded from further analysis.

Of the 271 eligible patients, 256 had been tested using the AlloSure dd-cfDNA assay (CareDx, Brisbane, CA, USA), while 15 had undergone testing with the Prospera assay (Natera, San Carlos, CA, USA). The study was approved by the Institutional Review Board (IRB) under the Ethical Oversight Submission System (ETHOS) at the University of Minnesota (approval code: STUDY00020719).

**Figure 1:**
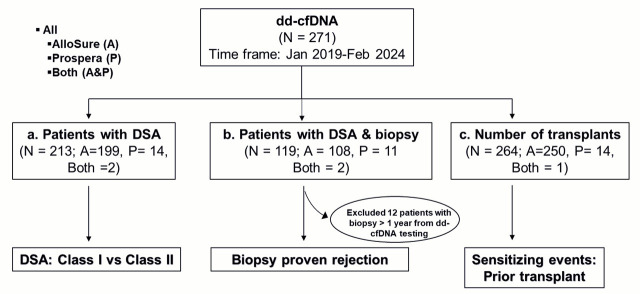
Flowchart of data collection and analysis: Kidney and pancreas transplant recipients who underwent dd-cfDNA testing between January 2019 and February 2024 were included. For analysis, patients with (**a**) donor-specific antibodies (DSA), (**b**) DSA and biopsy, and (**c**) a history of prior transplants were evaluated to compare dd-cfDNA levels in the context of DSA class (Class I vs. Class II), biopsy-proven rejection, and the potential impact of prior allograft exposure. AlloSure (A), Prospera (P), Both AlloSure, and Prospera (Both).

Subsequent stratification of the cohort was performed based on the availability of complementary test results (Figure 1). A total of 213 patients had DSA results available within six months of the dd-cfDNA test. Among these, 199 patients had been tested using AlloSure, 14 with Prospera, and 2 by both assays. A separate subgroup of 107 patients had DSA and biopsy results available within one year of the dd-cfDNA test. This subset included 96 patients tested with AlloSure, 11 with Prospera, and 2 with both assays. Additionally, 264 patients had dd-cfDNA results alongside documentation of the number of prior transplants, allowing for analysis of potential associations between dd-cfDNA levels and sensitizing events, particularly MOT recipients.

To investigate closer-in-time correlations, a focused analysis was also performed on patients with biopsy and DSA results obtained within six months of the dd-cfDNA test date.

### 2.2. Demographic Description

The entire study cohort (N = 271) was analyzed for demographic variables including gender, race, age, transplant organ type, and donor type. These characteristics are summarized in Table 1.

### 2.3. HLA Antibody Screening and Histocompatibility Testing

DSA were characterized using LABScreen Single Antigen Beads (One Lambda; West Hills, CA, USA) according to the manufacturer’s instructions. To mitigate the prozone effect, sera were either heat-inactivated (10 min at 56 °C) and treated to a final 1% fetal calf serum (FCS) concentration or treated to a final concentration of 22.5 mM EDTA and 1% FCS. HLA antibody strength was assessed using normalized mean fluorescence intensity (MFI). DSA positivity was defined per institutional protocols, with an MFI threshold greater than 500.

### 2.4. Donor-Derived Cell-Free DNA (dd-cfDNA) Assay

Venous blood for dd-cfDNA testing was collected in Streck Cell-Free DNA BCT tubes and stored at room temperature. For AlloSure testing, samples were shipped to the Clinical Laboratory Improvement Amendments (CLIA)-certified laboratory at CareDx, Inc. (Brisbane, CA, USA), where cfDNA analysis was performed as previously described [12,19]. For Prospera, samples were processed at a CLIA-certified laboratory at Natera, Inc. (Austin, TX, USA), with cfDNA testing conducted as outlined in prior studies [20]. Briefly, plasma was separated, and cfDNA was extracted and quantified using a targeted next-generation sequencing (NGS) assay, which accurately measures dd-cfDNA without requiring prior genotyping of the recipient or donor.

### 2.5. Biopsy

For this study, diagnoses were based on documented histological findings in patients’ medical records. Briefly, tissue samples, preserved in formalin and embedded in paraffin, were analyzed for histomorphologic features and C4d staining. Classification of rejection was performed by the institution’s board-certified pathologists and followed the Banff criteria, incorporating key histological findings such as glomerulitis (g), peritubular capillaritis (ptc), transplant glomerulopathy (cg), and C4d positivity, in addition to ultrastructural evidence like peritubular capillary basement membrane multilayering, and serologic detection of DSA.

### 2.6. Statistical Analysis

Comparative analyses were conducted using GraphPad Prism version 5.04 (GraphPad Software, La Jolla, CA) to assess concordance between dd-cfDNA test results and established indicators of transplant rejection, such as DSA and biopsy findings. Dd-cfDNA results were analyzed independently for each assay type (AlloSure and Prospera) and in aggregate. Due to the small sample size (especially in the Prospera group), the Mann–Whitney U test was used for pairwise comparisons as normality could not be reliably assessed. For comparisons across multiple groups, the non-parametric Kruskal–Wallis test followed by Dunn’s post hoc test was applied. A *p*-value of less than 0.05 was considered statistically significant.

### 2.7. Evaluation of Diagnostic Test Utility

To evaluate the diagnostic performance of dd-cfDNA testing, results from both the AlloSure and Prospera assays were compared to biopsy findings, using a threshold of 1% dd-cfDNA to define a positive result, as recommended by the respective vendors. Diagnostic utility was quantified through calculations of clinical sensitivity, specificity, positive predictive value (PPV), and negative predictive value (NPV). Receiver operating characteristic (ROC) curve analysis was performed by plotting sensitivity against 1-specificity for each assay.

## 3. Results

### 3.1. Study Demographic

The study population consisted of 58% males, 41.6% females, and 0.4% individuals with unknown gender identity. Racial composition included 61.5% Caucasian, 16.8% African American, and 10.7% Asian individuals. Participants ranged in age from 1 to 78 years, with an average age of 53.4 years. Most organ transplants were renal (92.7%), with kidney-pancreas (KP) and kidney-liver (KL) transplants making up 2.7% and 1.5%, respectively, of the remaining 7%. Approximately 60% of the transplants originated from deceased donors, while 40% came from living donors (Table 1).

### 3.2. Dd-cfDNA Is Elevated in Patients with DSA

**Figure 2:**
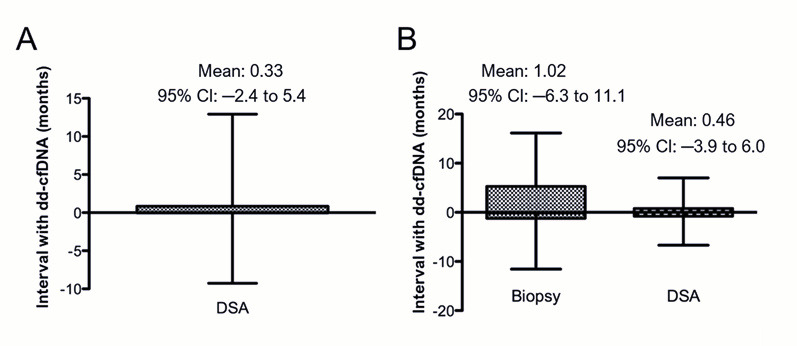
Time intervals between tests: (**A**) Patients with dd-cfDNA and DSA within 6 months of each other (N = 213: AlloSure, n = 199; Prospera, n = 14; Both AlloSure and Prospera, n = 2). (**B**) Patients who had all three tests (dd-cfDNA, biopsy, and DSA) within one year of each other (N = 107: AlloSure, n= 96; Prospera, n = 11; Both AlloSure and Prospera, n = 2).

**Figure 3:**
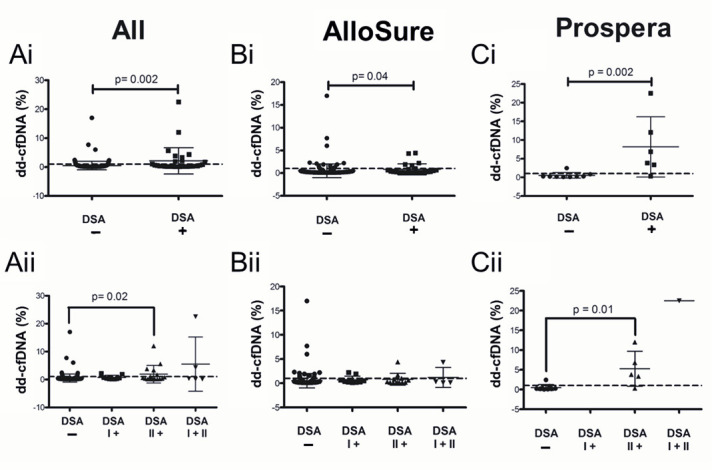
Dd-cfDNA levels in the presence of donor-specific antibodies (DSA) testing. Comparison of dd-cfDNA levels in patients (i) Without DSA vs. with DSA and (ii) stratified by the detected DSA class when dd-cfDNA was detected by (**A**) Both AlloSure and Prospera (All), (**B**) AlloSure only, and (**C**) Prospera only. The dotted line indicates the 1% dd-cfDNA threshold. N = 119: AlloSure, n = 108; Prospera, n = 11; Both AlloSure and Prospera, n = 2. For pairwise comparisons the Mann–Whitney U test was used. For comparisons across groups, Kruskal–Wallis non-parametric test followed by the Dunn’s post-hoc test was performed. A *p*-value < 0.05 is considered statistically significant. Mean dd-cfDNA (**Ai**) All: 0.53% DSA−, 2.15% DSA+; (**Bi**) Allosure: 0.52% DSA−, 0.82% DSA+; (**Ci**) Prospera: 0.50% DSA−, 8.14% DSA+; (**Aii**) All: 0.53% DSA−, 0.71% DSA I+; 1.91% DSA II+, 5.47% DSA I+II; (**Bii**) Allosure: 0.52% DSA−, 0.71% DSA I+; 0.82% DSA II+, 1.21% DSA I+II; (**Cii**) Prospera: 0.5% DSA−, 0.0% DSA I+; 5.27% DSA II+, 22.52% DSA I+II.

The mean time interval between dd-cfDNA and DSA testing was 0.33 months (95% CI:−2.4–5.4 months; Figure 2A). The mean interval between dd-cfDNA and biopsy was 1.02 months (95% CI:−6.3–11.1 months), while the mean interval between dd-cfDNA and DSA testing in patients who underwent concomitant biopsies was 0.46 months (95% CI:−3.9–6.0 months; Figure 2B). Significantly elevated levels of dd-cfDNA were observed in DSA-positive compared to DSA-negative patients. This difference was evident when analyzed across the entire cohort (*p* = 0.002; Figure 3Ai) as well as within subsets tested by AlloSure (*p* = 0.04; Figure 3Bi) or Prospera (*p* = 0.002; Figure 3Ci). When stratifying the full dataset by DSA class (Class I, Class II, or both), dd-cfDNA levels remained significantly elevated in patients with Class II DSA only (*p* < 0.05; Figure 3Aii), with a particularly strong association observed in the Prospera group (*p* = 0.01; Figure 3Cii). No significant correlation was noted between DSA class and dd-cfDNA levels in the AlloSure group (Figure 3Bii). Correlation analysis revealed a stronger relationship between DSA MFI and dd-cfDNA levels in samples tested with Prospera (r^2^ = 0.71; Supplemental Figure S1C), compared to AlloSure (r^2^ = 0.04; Supplemental Figure S1B) or the combined dataset (r^2^ = 0.18; Supplemental Figure S1A).

### 3.3. Dd-cfDNA Levels Show Limited Correlation with Biopsy-Proven Rejection

**Figure 4:**
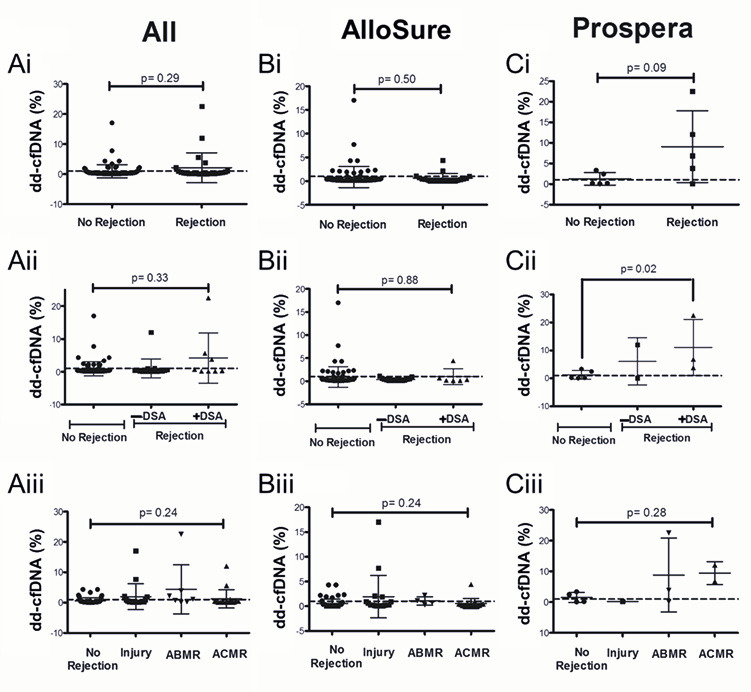
Dd-cfDNA levels within one year of biopsy evaluation: Comparison of dd-cfDNA levels in patients with no rejection vs. (i) Biopsy-proven rejection, (ii) Rejection with or without DSA and (iii) Injury, ABMR, and ACMR, with dd-cfDNA from (**A**) Both AlloSure and Prospera, (**B**) AlloSure only, and (**C**) Prospera only. The dotted line indicates a dd-cfDNA threshold of 1%. N = 107: AlloSure, n = 96; Prospera, n = 11; Both AlloSure and Prospera, n = 2. For pairwise comparisons the Mann–Whitney U test was used. For comparisons across groups, Kruskal–Wallis non-parametric test followed by the Dunn’s post-hoc test was performed. A *p*-value < 0.05 is considered statistically significant. Antibody mediated rejection (ABMR), Acute cellular rejection (ACMR). Mean dd-cfDNA (**Ai**) All: 0.90% No Rejection, 2.12% Rejection; (**Bi**) AlloSure: 0.87% No Rejection, 0.63% Rejection; (**Ci**) Prospera: 1.24% No Rejection, 9.06% Rejection; (**Aii**) All: 0.90% No Rejection, 1.03% Rejection −DSA, 4.18% Rejection +DSA; (**Bii**) AlloSure: 0.87% No Rejection, 0.36% Rejection −DSA, 0.98% Rejection +DSA; (**Cii**) Prospera: 1.24% No Rejection, 6.07% Rejection −DSA, 11.06% Rejection +DSA; (**Aiii**) All: 0.61% No Rejection, 1.95% Injury, 4.39% ABMR, 1.24% ACMR; (**Biii**) AlloSure: 0.55% No Rejection, 1.95% Injury, 1.07% ABMR, 0.53% ACMR; (**Ciii**) Prospera: 1.51% No Rejection, 0.17% Injury, 8.81% ABMR, 9.43% ACMR.

Although not statistically significant, the overall dataset demonstrated higher dd-cfDNA levels in the rejection group compared to the no-rejection group. Among patients with biopsy-proven rejection, those with DSA exhibited significantly elevated dd-cfDNA levels when tested using the Prospera assay within one year of DSA detection (*p* = 0.02; Figure 4Cii). However, this association was not observed with the AlloSure assay or in broader analyses that included all biopsy/DSA data collected within either one year or six months of dd-cfDNA testing (Figure 4; Supplemental Figure S2).

### 3.4. Multi-Organ Transplants Elevate dd-cfDNA in a Vendor-Specific Manner

**Figure 5:**
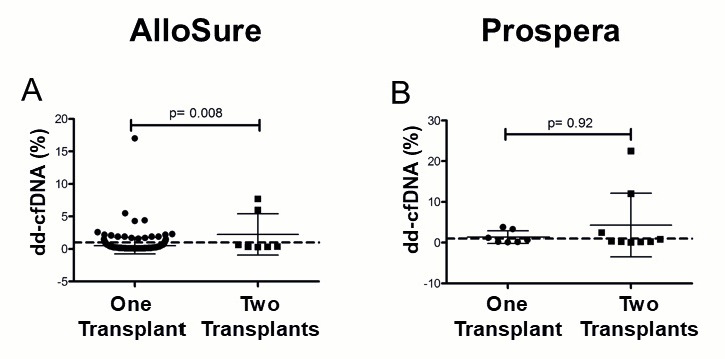
Dd-cfDNA levels in multi-graft recipients in (**A**) AlloSure, n = 250; (**B**) Prospera, n = 14. The Mann–Whitney U test was performed to statistically determine the significance of the difference. *p* < 0.05 is statistically significant. Mean dd- cfDNA: (**A**) AlloSure: 0.52% One transplant, 3.01% two transplants; (**B**) Prospera: 1.36% One transplant, 3.84% two transplants. The dotted line indicates the 1% dd-cfDNA threshold.

Patients with a history of multi-organ transplantation (MOT) demonstrated significantly elevated dd-cfDNA levels in the AlloSure group (*p* = 0.008), whereas no significant difference was observed in the Prospera group (Figure 5). In the AlloSure cohort (n = 250), there were 7 MOT recipients: 1 kidney-pancreas (KP) and 2 kidney-liver (KL), 2 kidney-heart (KH), and 2 kidney-lung (KLu). Rejection or DSA was not detected in 6 of these patients; however, 1 kidney-heart recipient had a low-level DSA (MFI = 571), and biopsy data was unavailable. In the Prospera cohort (n = 15), there were 9 MOT recipients: 6 KP, 2 KL, and 1 kidney-heart (KH). No rejection or DSA was identified in these patients, except for 2 (1 KP and 1 KL) who lacked DSA testing, and 1 kidney-pancreas recipient for whom biopsy data were unavailable. These findings suggest that sensitizing events, such as prior transplantation, may impact dd-cfDNA measurements in a vendor-dependent manner.

### 3.5. Diagnostic Utility Varies Between dd-cfDNA Vendors

Using a 1% threshold, diagnostic utility differed between the two dd-cfDNA platforms. AlloSure showed high specificity (80%) but low sensitivity (19%), with a negative predictive value (NPV) of 77% and positive predictive value (PPV) of 21%. In contrast, Prospera demonstrated higher sensitivity (75%) and moderate specificity (60%), with an NPV of 75% and PPV of 60% (Supplemental Table S1). The area under the curve (AUC) for both vendors were comparable: 0.86 (95% CI: 0.83–0.94) and 0.80 (95% CI: 0.61–0.99) for AlloSure and Prospera respectively (Supplemental Figure S3). Hence the clinical utility of the dd-cfDNA tests could vary with the assay type represented by two different vendors here.

## 4. Discussion

This study assessed the performance of dd-cfDNA assays from two commercial vendors in a cohort primarily composed of males (58%) and Caucasian (61%) patients, with a mean age of 53 years. The majority were renal transplant recipients (93%), the most of whom received grafts from deceased donors (59%). Elevated dd-cfDNA levels were strongly associated with donor-specific antibody (DSA) positivity, particularly among patients with HLA class II DSA. We also identified vendor-specific differences between dd-cfDNA and biopsy-proven rejection in DSA-positive patients, as well as higher dd-cfDNA levels in individuals with multi-organ transplants. Remarkably, the diagnostic utility of dd-cfDNA varied between vendors. Although dd-cfDNA assays have been widely studied [2,16,18,21], the specific applicability within our cohort has not been well established, underscoring the significance of our findings.

Our study is distinctive in that it retrospectively analyzed provider-initiated, send-out dd-cfDNA test results performed by commercial reference laboratories, in contrast to most transplant-related testing otherwise performed “in-house”. Unlike prospective dd-cfDNA studies, which typically benefit from rigorous and standardized sample collection protocols, our real-world analysis reflects organically ordered tests without controlled timing. Despite this inherent limitation, we evaluated the temporal proximity between dd-cfDNA and associated clinical tests. The mean time difference between dd-cfDNA and biopsy was 1.02 months (95% CI: −6.3 to 11.1 months), and 0.46 months (13.8 days) (95% CI: −3.9 to 6.0 months) between dd-cfDNA and DSA. These findings suggest no substantial time gaps between assays, supporting the validity of our concordance analysis. A key challenge addressed in our study is the difficulty of correlating diagnostic tests, such as dd-cfDNA, DSA testing, and biopsy, which are infrequently performed simultaneously in routine clinical care. We observed that the interval between dd-cfDNA and DSA testing could extend up to six months, and up to one year between dd-cfDNA testing and biopsy. Additionally, cfDNA testing in our cohort was performed by external vendors, further introducing potential variability. These factors highlight the complexities and confounding variables present in real-world clinical practice, which should be considered when interpreting dd-cfDNA results.

Few studies have examined dd-cfDNA in DSA-positive patients [19,22]. Our data indicates that dd-cfDNA may serve as an adjunctive tool in such cases. Specifically, we observed elevated dd-cfDNA levels in patients with HLA class II DSA, particularly in those tested with the Prospera assay. Despite the smaller cohort size in the Prospera group, which we address by utilizing non-parametric statistics, our results are consistent with previous reports highlighting improved diagnostic accuracy of dd-cfDNA in detecting antibody-mediated rejection (ABMR) in DSA-positive individuals [22]. Our analysis revealed no meaningful correlation between dd-cfDNA levels and histologically confirmed rejection (ABMR, Acute cellular rejection-ACMR, or other forms of injury) within both six- and twelve-month intervals. This may be due to temporal mismatches between increase in dd-cfDNA levels and biopsy testing. While dd-cfDNA shows promise in identifying active ABMR [18,23] or increased levels observed in patients with DSA [19,22], particularly in patients with class II DSA, which is associated with poorer graft outcomes [24,25,26], it does not reliably predict biopsy-proven rejection on its own. The combination of elevated dd-cfDNA and the presence of DSA offers more compelling non-invasive justification for performing a biopsy than either DSA or dd-cfDNA alone. Current vendor recommendations support using dd-cfDNA testing in conjunction with other clinical and laboratory findings to guide transplant surveillance.

Several studies have established dd-cfDNA as a reliable non-invasive biomarker for allograft rejection, with negative predictive values exceeding 90% in some cases [9] highlighting a strong potential to reduce reliance on invasive biopsies. The reported area under the curve (AUC) for distinguishing ABMR from non-rejection ranges from 0.81 to 0.91% [22,23], with a pooled AUC of 0.89 based on a meta-analysis of five studies [27]. Our findings suggest that clinical utility of dd-cfDNA varies by assay vendor. At a 1% threshold, both AlloSure and Prospera assays showed diagnostic performance consistent with published data. AlloSure demonstrated higher specificity (80%) but lower sensitivity (19%), favoring its role in ruling out rejection. In contrast, Prospera showed higher sensitivity (75%) and lower specificity (60%), supporting its use for early detection. Variations between our study and prior studies may reflect differences in study design, including biopsy-matched sampling and cohort size. For instance, Sigdel et al. reported an AUC of 0.87, with a sensitivity of 88.7% and specificity of 72.6%, using the Prospera assay at a 1% threshold to distinguish active rejection from non-rejection [28].

We show that patients with two functioning grafts tend to have higher levels of dd-cfDNA when tested by the AlloSure assay. This finding aligns with prior observations in lung [21,29] and heart transplantation [30], where dd-cfDNA levels were lower in single lung transplant recipients compared to those with double lung transplants, even in clinically stable patients, suggesting that donor mass can influence dd-cfDNA levels. Notably, this difference was resolved when dd-cfDNA levels in single lung recipients were adjusted by doubling the values, both at baseline and during episodes of allograft injury [29]. Our study and others highlight the importance of accounting for total donor tissue mass when interpreting dd-cfDNA values in clinical practice.

Although both assays use NGS to quantify single nucleotide polymorphisms (SNPs) in dd-cfDNA, differences in performance may stem from assay design and the diagnostic thresholds employed. AlloSure targets 405 SNPs across 22 somatic chromosomes [12,31], while Prospera analyzes 13,392 SNPs across four chromosomes [28]. In fact, in a study involving 76 patients, the Prospera assay yielded higher dd-cfDNA values at the 1% threshold, consistent with our findings. However, at a 0.5% threshold, AlloSure outperformed Prospera in detecting cell-mediated rejection (CMR) [31,32]. Optimal dd-cfDNA cutoff values may vary by study and clinical context. For example, a custom assay recommended 0.75% for renal transplant monitoring [7]; AlloSure demonstrated strong performance at 0.74% for distinguishing ABMR from CMR [18], and 0.5% was found to be optimal for detecting CMR in a cohort of 79 biopsy-confirmed cases [32]. Another custom assay using a 1% threshold across 300 plasma samples achieved 87% sensitivity and 73% specificity for detecting transplant rejection [33]. These findings underscore the importance of establishing institution-specific diagnostic thresholds tailored to patient populations.

In summary, these findings underscore the importance of establishing context-specific applications for dd-cfDNA assays. For laboratories or institutions considering in-house implementation—especially given that most current dd-cfDNA assays are laboratory-developed tests—it is essential to define diagnostic thresholds tailored to the transplanted organ. Special consideration should be given to patients with prior sensitizing events, particularly those who have undergone multi-organ transplantation. In such cases, monitoring longitudinal dd-cfDNA trends relative to baseline values may provide more meaningful insights. Additionally, if grafts are from different donors, it may be preferable to utilize assays that include markers capable of distinguishing between individual donors. As the field continues to evolve, effective clinical integration of these assays will require a comprehensive understanding of their underlying technologies, benefits, and limitations—especially as new data and platforms emerge.

## 5. Conclusions

This study evaluated the utility of two dd-cfDNA assays in our patient population over a five-year period, comparing their performance against DSA and biopsy results. Our findings highlight the vendor-specific correlations including stronger associations with biopsy-proven rejection in DSA-positive patients and elevated dd-cfDNA levels in multi-organ transplant recipients. Additionally, the clinical utility of each test may vary, with specific applications suited to different transplant populations.

## Figures and Tables

**Table 1:** Demographic Characteristics of the Study Cohort: Kidney-pancreas (KP), kidney-liver (KL), kidney-lung (KLu), kidney-heart (KH). The sex, race, age (in years), organ type (%), and donor type (%) are tabulated.

Demographic DSA (n = 271)									
Race (%)		Sex (%)		Age (Years)		Organ Type (%)		Donor Type (%)	
White/Caucasian	61.5	Male	58	Mean	53.4	Kidney	92.7	Living	40.5
Black/African American	16.8	Female	41.6	Minimum	1.3	Pancreas	1.1	Deceased	59.5
Hispanic/Latino	1.9	Other	0.4	Maximum	78.4	KP	2.7		
Asian	10.7					KL	1.5		
Middle Eastern	0.4					KLu	0.7		
Indigenous American/ Alaskan Native	1.9					KH	1.1		
Mixed	5.7								
Other	1.1								

## Data Availability

The original contributions presented in this study are included in the article/Supplementary Materials. Further inquiries can be directed to the corresponding author.

## Supplementary Materials

The following supporting information can be downloaded at: https://www.mdpi.com/article/10.3390/genes16070724/s1, Supplemental Figure S1: Regression analysis evaluating the relationship between dd-cfDNA levels and DSA mean fluorescence intensity (MFI), Supplemental Figure S2: Dd-cfDNA levels within 6 months of biopsy evaluation, Supplemental Table S1: Diagnostic performance of dd-cfDNA tests compared to biopsy finding, Supplemental Figure S3: Diagnostic accuracy of cfDNA assays.

## Abbreviations

MOT	Multi-organ transplantation
Dd-cfDNA	Donor-derived cell-free DNA
DSA	Donor-specific antibody
NGS	Next-generation sequencing
ABMR	Antibody mediated rejection
ACMR	Acute cellular rejection
AUC	Area under the curve
SNPs	Single nucleotide polymorphisms
