# Supplementary material for: Evaluating the Diagnostic Utility of dd-cfDNA in Renal Allograft Surveillance: A Single-Center Perspective
Source: Genes (Basel). 2025 Jun 21;16(7):724. doi: 10.3390/genes16070724 (PMC12294478; doi:10.3390/genes16070724)
Supplement: Supplementary file 1 [file genes-16-00724-s001.zip › genes-3677500-supplementary.pdf]

Evaluating the diagnostic utility of dd-cfDNA in renal allograft surveillance: a single-center perspective

Supplemental Figures and Tables

All AlloSure Prospera

S1A S1B S1C

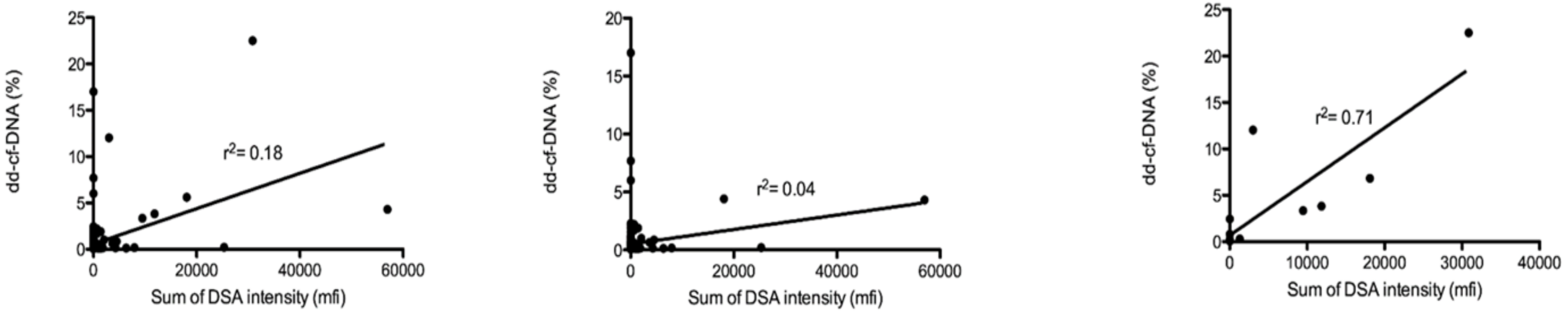

Supplemental Figure S1: Regression analysis evaluating the relationship between dd-cfDNA levels and DSA mean fluorescence intensity (MFI) when dd-cfDNA was detected by (3A) Both AlloSure and Prospera (All), (3B) AlloSure only, and (3C) Prospera only. N=119: AlloSure, n=108; Prospera, n=11; Both AlloSure and Prospera, n=2.

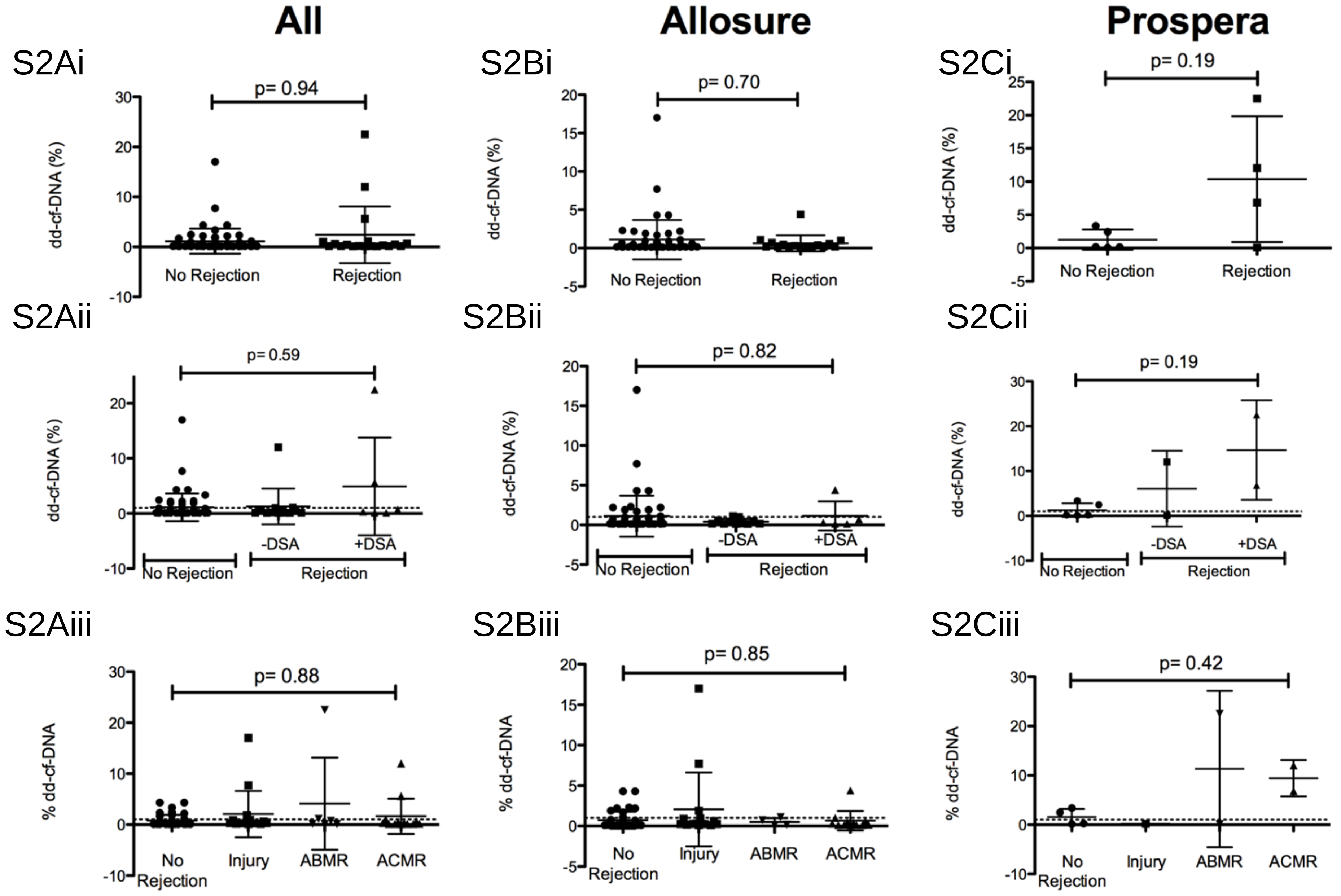

Supplemental Figure S2: Median dd-cfDNA levels within 6 months of biopsy evaluation: Comparison of dd-cfDNA levels in patients with no rejection vs. S2i) Biopsy-proven rejection, S2ii) Rejection with or without DSA and S2iii) Injury, ABMR, and ACMR, with dd-cfDNA from (S2A) Both AlloSure and Prospera (All), (S2B) AlloSure only, and (S2C) Prospera only. The dotted line indicates a Dd-cfDNA threshold of 1%. N=77: AlloSure, n=70; Prospera, n=9; Both AlloSure and Prospera, n=2. For pairwise comparisons the Mann-Whitney U test was used. For comparisons across groups, Kruskal-Wallis non-parametric test followed by the Dunn’s post-hoc test was performed. A p-value < 0.05 is considered statistically significant.

Supplemental Table S1: Diagnostic performance of dd-cfDNA tests compared to biopsy finding: S. Table 1A) AlloSure, n=96 and S. Table 1B) Prospera, n=11. Biopsy results were evaluated against dd-cf-DNA at a >1% threshold to calculate the diagnostic characteristics of each assay.

Table S1A

**AlloSure Values**	**Fraction**	**%**
Sensitivity	0.188	19
Specificity	0.796	80
PPV	0.214	21
NPV	0.768	77

Table S1B

**Prospera Values**	**Fraction**	**%**
Sensitivity	0.75	75
Specificity	0.6	60
PPV	0.6	60
NPV	0.75	75

S3A S3B

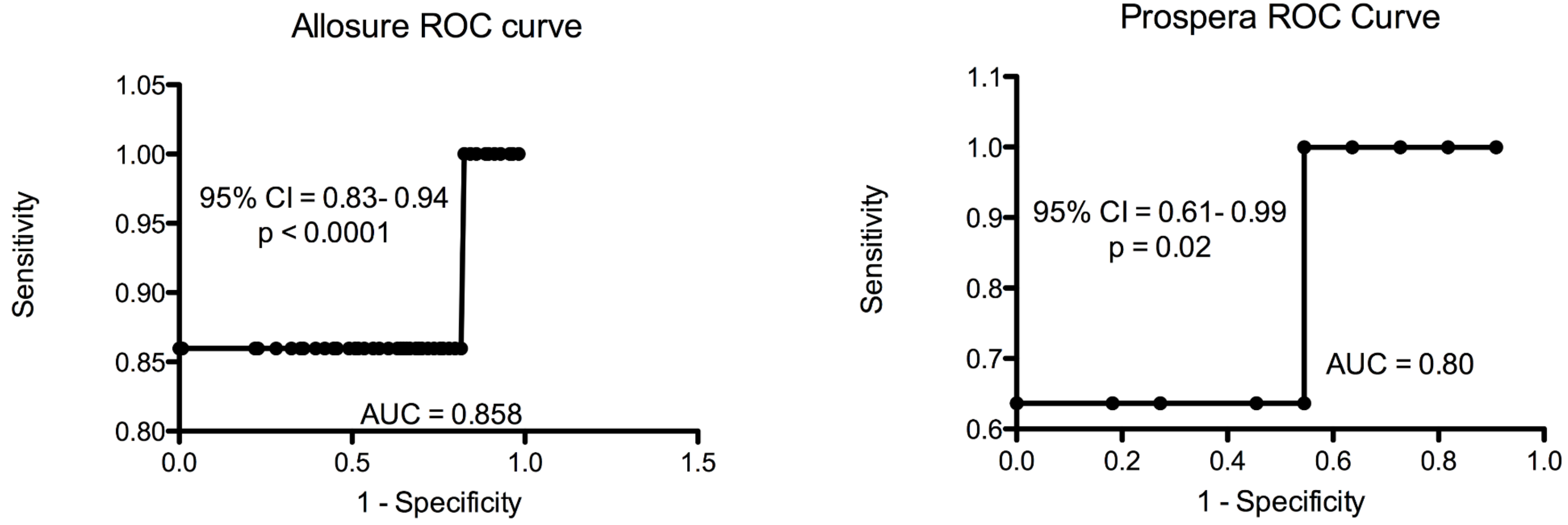

Supplemental Figure S3. Diagnostic accuracy of cfDNA assays. The receiver-operating-characteristic analysis showing the fraction of true positive results (sensitivity) and the fraction of false positive results (1-specificity) of S3A) AlloSure and S3B) Prospera . Biopsy results were compared with dd-cf-DNA results at >1% threshold for discriminating biopsy-proven rejection from no rejection Sample size: N=107: AlloSure, n=96; Prospera, n=11; Both AlloSure and Prospera, n= 2.
